# A patient with superior semicircular canal dehiscence presenting with Tullio's phenomenon: a case report

**DOI:** 10.1186/1752-1947-3-22

**Published:** 2009-01-23

**Authors:** Richard JD Hewitt, Anthony O Owa

**Affiliations:** 1 Department of Otolaryngology, Queen's Hospital, Rom Valley Way, Romford, Essex, RM7 0AG, UK

## Abstract

**Introduction:**

Superior semicircular canal dehiscence represents a manageable cause of sound and pressure induced vertigo. This case highlights its presentation and investigation, including a review of the literature, and the authors' surgical technique used in its successful treatment.

**Case presentation:**

A 45-year-old Caucasian man presented with vertigo induced by sound or pressure. Subsequent investigation revealed dehiscence of the superior semicircular canal and the patient underwent a surgical repair.

**Conclusion:**

Surgery to repair or resurface the dehiscence represents an effective treatment modality, offering a resolution of symptoms with no detrimental effect on hearing or long-term sequelae. A five-layer composite repair consisting of temporalis fascia – bone pate – conchal cartilage – bone pate – temporalis fascia has been found to be safe and effective.

## Introduction

Dehiscence of bone overlying the superior semi-circular canal was described in 1998 by Minor *et al*. [1] as a cause of sound and pressure induced vertigo. The condition of superior semicircular canal dehiscence has subsequently been the topic of numerous articles exploring the clinical presentation, investigation and management of the disorder. The incidence of dehiscent bone has been reported in cadaveric analysis to lie between 0.4 and 0.5%, with thinning of the bone to <0.1 mm in a further 1.4% [2].

Symptoms include one or more of the following: sound induced vertigo, often in a vertical-torsional plane; conductive hyperacusis; and chronic feelings of disequilibrium and motion intolerance [3]. Clinical evaluation with a patient exposed to sound or pressure, wearing Frenzel's glasses, reveals nystagmus of an upward and anticlockwise nature in a right-sided lesion, and upward and clockwise in a left-sided lesion [2]. Radiological imaging, with high resolution computed tomograms of the temporal bones, has a high sensitivity for the diagnosis of superior semicircular canal dehiscence but needs to be correlated with patient history, clinical examination and audiological and vestibular assessment to achieve a high specificity.

The treatment is either conservative, with the avoidance of causative stimuli, or surgical, if the symptoms are uncontrollable. Surgical repair or resurfacing of the dehiscence area of bone is the recommended interventional approach. There have, however, been many proposed approaches, materials and techniques. However, it is agreed that surgery can result in complete resolution of symptoms in most patients [1]. The surgical technique has been described with various resurfacing methods including three- and five-layer techniques. This article describes a surgical approach using a five-layer technique for the repair of the dehiscence conducted in a district general hospital with complete resolution of symptoms and no detrimental effects on hearing and no long-term sequelae [4].

## Case presentation

**Figure 1:**
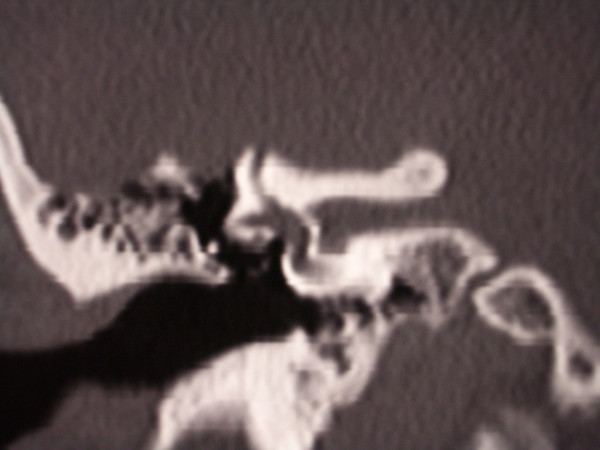
**Pre-operative temporal bone coronal computed tomography images**.

A 45-year-old Caucasian patient presented with a 5-year isolated history of noise induced vertigo and a history of head trauma 12 years ago. A Tullio test revealed the appropriate nystagmus, when presented with a noise of -100 dB at 1000 Hz, to indicate a right-sided lesion. Pure tone audiometry revealed a bilateral, symmetrical, sensorineural hearing loss of 25 dB with a dip to 70 dB at 4000 Hz. High resolution computerised tomography imaging demonstrated both superior semi-circular canals to be dehiscent and no other abnormalities (Figure 1). The patient was counselled as to the treatment options and elected to have a surgical repair to the symptomatic right side via a middle fossa approach.

The surgery was conducted in conjunction with the neurosurgeons and involved the harvesting of conchal cartilage, temporalis fascia and bone pate. The dura was elevated from the dehiscent semicircular canal and the dehiscent tegmen resurfaced with a five-layer composite consisting of temporalis fascia – bone pate – conchal cartilage – bone pate – temporalis fascia. The procedure went without complication.

**Figure 2:**
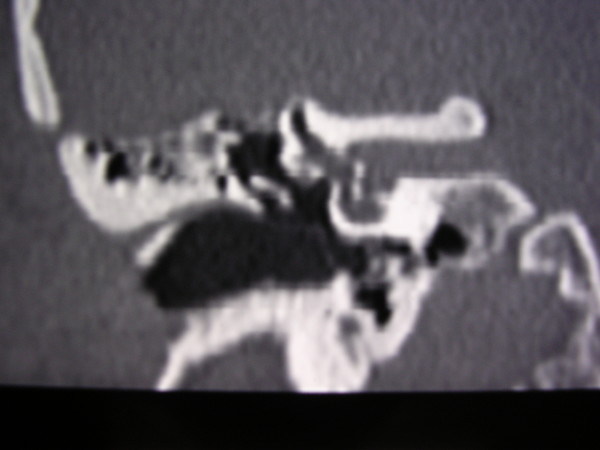
**Postoperative temporal bone coronal computed tomography images**.

Subsequent consultation, up to 18 post-operation, revealed a total resolution of noise induced symptoms. Tullio testing produced no nystagmus, pure tone audiometry demonstrated no change in the hearing threshold and computerised tomography imaging demonstrated an intact, right-sided semi-circular canal (Figure 2).

## Conclusion

The authors advocate the use of a five-layer composite repair, consisting of temporalis fascia – bone pate – conchal cartilage – bone pate – temporalis fascia, via a middle fossa approach to repair or resurface symptomatic dehiscent semicircular canals. This is a safe and effective method with no side effects in the short to medium term.

## Consent

Written informed consent was obtained from the patient for publication of this case report and any accompanying images. A copy of the written consent is available for review by the Editor-in-Chief of this journal.

## Competing interests

The authors declare that they have no competing interests.

## Authors' contributions

AO and RH undertook the management, writing and approval of the manuscript.
